# Crosstalk between Iron Metabolism and Erythropoiesis

**DOI:** 10.1155/2010/605435

**Published:** 2010-06-10

**Authors:** Huihui Li, Yelena Z. Ginzburg

**Affiliations:** Lindsley F. Kimball Research Institute, New York Blood Center, 310 East 67th Street, 1–38, NY 10065, USA

## Abstract

Iron metabolism and erythropoiesis are inextricably linked. The majority of iron extracted from circulation daily is used for hemoglobin synthesis. In the last 15 years, major advances have been made in understanding the pathways regulating iron metabolism. Hepcidin is a key regulator of iron absorption and recycling and is itself regulated by erythropoiesis. While several viable candidates have been proposed, elucidating the “erythroid regulator” of hepcidin continues to generate significant experimental activity in the field. Although the mechanism responsible for sensing iron demand for erythropoiesis is still incompletely understood, evaluating diseases in which disordered erythropoiesis and/or iron metabolism are showcased has resulted in a more robust appreciation of potential candidates coordinated erythroid iron demand with regulators of iron supply. We present data drawn from four different conditions—iron deficiency, congenital hypotransferrinemia, beta-thalassemia, and hereditary hemochromatosis—both in human and non-human models of disease, together suggesting that erythroid iron demand exerts a stronger influence on circulating iron supply than systemic iron stores. Greater understanding of the interplay between the key factors involved in the regulation of iron metabolism and erythropoiesis will help develop more effective therapies for disorders of iron overload, iron deficiency, and hemoglobin synthesis.

## 1. Introduction

Iron is an essential element for almost all living organisms, from mammals and lower vertebrates down to unicellular organisms. It forms the core of molecules such as hemoglobin and myoglobin and is necessary for cytochrome production. Two to three million red blood cells (RBCs) are produced every second and require 30–40 mg of iron delivered to the erythron to make 30 pg of hemoglobin per cell, a total of 6 g of hemoglobin daily. The pool of iron bound to transferrin [Tf-Fe(III)] is 10 times smaller than the daily iron requirements, requiring rapid turn around to ensure sufficient delivery of iron. Daily iron required for erythropoiesis is predominantly derived from recycling of heme iron by macrophages erythrophagocytosing senescent RBCs. Because the majority of iron in most organisms can be found in the hemoglobin compartment, erythropoiesis dominates iron metabolism and the two are inextricably intertwined. This dynamic process of iron trafficking for erythropoiesis requires significant crosstalk to prevent iron deficiency or iron overload and protect the organism from developing anemia as well as from the potential toxicity of excess iron. For instance, iron absorption increases, often dramatically, when erythropoiesis occurs at a higher than normal rate to accommodate the higher iron demand. Chronic blood loss, for example, results in both a maximal stimulation of iron absorption and less iron per cell leading to smaller RBCs with less hemoglobin before progressing to decreased RBC counts and anemia. Conversely, disease states of excess iron are often associated with expanded RBC size and higher cellular hemoglobin concentrations as a way of sequestering iron into a nontoxic compartment. Lastly, diseases in which anemia and excess iron coexist exhibit complicated regulation schema that are still incompletely understood.

Iron directed to the erythroid compartment is restricted to transferrin-bound iron (Tf-Fe(III)) and its ability to bind transferrin receptor (TfR1) is a well worked out paradigm. Transferrin is the second most abundant serum protein, after albumin, and takes up iron from duodenal enterocytes when it is absorbed and from macrophages when iron is recycled from senescent RBCs. Iron absorption and recycling are regulated by hepcidin, a peptide hormone thought to be the main regulator of iron flows in the body. Hepcidin exerts its function by binding to the only known iron export protein, ferroportin (FPN-1), found on hepatocytes, macrophages, duodenal enterocytes, and placental cells, all involved in iron metabolism. Hepcidin binds FPN-1, causes its internalization and degradation, and results in cessation of iron release from cells [1]. Regulation of hepcidin has been extensively studied in recent years. It is known that hepcidin is regulated by iron, hypoxia, inflammation, and erythropoiesis. While evidence exists for an “erythroid regulator” of hepcidin, the mechanisms by which this is accomplished are still under investigation.

Understanding the crosstalk between iron regulation and erythroid proliferation and maturation is a hotbed of research activity by multiple groups from many different angles. Much can be learned from various disease states and experimental models of disease. This review attempts to catalog some of these for the purpose of elucidating the current state of knowledge on this subject.

## 2. Iron Metabolism, Iron Deficiency, and Anemia

Iron deficiency anemia (IDA) is one of the most common diseases worldwide and is typically associated with a microcytosis and hypochromasia. The presence of small (low MCV) and pale (low MCH) RBCs is typically indicative of low cellular hemoglobin and results from defects in heme or globin synthesis. While defects in globin synthesis are typically caused by genetic defects (e.g., *α*- and *β*- thalassemia), heme synthesis defects most commonly result from iron deficiency. In the pediatric population, this later manifestation results from nutritional iron deficiency, while in adults, the cause is more likely blood loss. As a better understanding of erythropoiesis evolved in the middle of the last century, the ease of diagnosing and treating IDA resulted in a great deal of comfort on the part of clinicians. However, there are several clinical scenarios that showcase the complex relationship between iron supply, iron demand, and erythropoiesis. 

The amount of iron delivered to each erythroid precursor depends on the amount of monoferric and diferric transferrin found in circulation as well as the density of TfR1 on the cell surface. Typically, each erythroid precursor has over a million TfR1s on its membrane because of its large iron requirement—for hemoglobin synthesis—relative to all other cells in the body [2]. In IDA, TfR1 membrane density increases further [3, 4] and drives up the concentration of soluble TfR1. Soluble TfR1 is normally found in the serum as a truncated version of membrane-bound TfR1 in quantities proportional to the amount found on the cell surface [5, 6]. Increased density of membrane-bound TfR1 enables the cell to increase avidity for iron during iron deficiency. Increased TfR1 and soluble TfR1 levels can also be found during stress erythropoiesis when the normally ample amount of systemic iron may require supplementation to enable a higher rate of heme synthesis. 

The mechanism by which cells alter their TfR1 expression involves iron regulatory proteins (IRPs) which have a high affinity for iron response elements (IREs) present in the mRNA of target genes involved in iron homeostatis. If an IRE is on the 5′ untranslated region, mRNA is more likely to be degraded whereas if it is on the 3′ untranslated region, it is more likely to be stabilized as a consequence of IRP binding. Thus, in an iron depleted state, when IRPs are able to bind mRNA, ferritin mRNA with a 5′ untranslated region IRE is more likely to be degraded while TfR1 mRNA with a 3′ untranslated region IRE proceeds to translation more readily. IRP2 knockout mice develop microcytic hypochromic anemia probably as a result of the reduced TfR1 expression in erythroid precursors [7, 8]. As a consequence of IRE/IRP regulation, iron deficiency is associated with high TfR1 expression and low ferritin concentrations [9]. 

**Figure 1:**
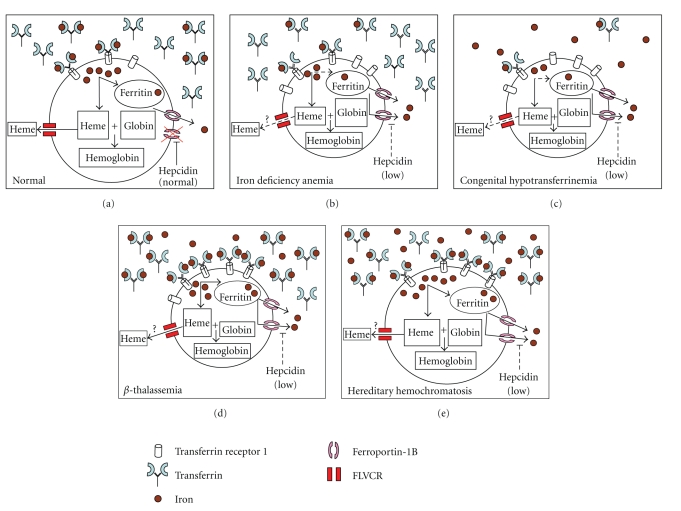
Model relationship between iron delivery, relative abilities to synthesize heme and globin, and heme and iron export in various diseases associated with concurrent iron and erythroid pathology.

Iron uptake starts when Tf-Fe(III) binds TfR1 (Figure 1). Under normal circumstances, the affinity of TfR1 for diferric transferrin is greater than for monoferric transferrin. However, this greater affinity wanes as the iron supply is diminished [10, 11]. Monoferric transferrin is the predominant form of transferrin in circulation when transferrin saturation is lowered [12]. The loss of preference for diferric transferrin may itself be a result of a proportionally higher concentration of monoferric transferrin, and relatively fewer diferric transferrin molecules, in circulation. Each molecule of monoferric transferrin delivers less iron to erythroid precursors than diferric transferring [10]. This enables a greater number of erythroid precursors to receive a smaller portion of the iron pool to offset the potential of developing anemia. This finding is consistent with the fact that MCV drops before hemoglobin decreases during the progressive worsening of iron deficiency. What controls this apportioning of iron to erythroid precursors is not completely understood.

Iron gains entry into erythroid precursors when Tf-Fe(III) complexes with TfR1 and is internalized into a clathrin-coated pit which matures into an endosome with a lowered pH, facilitating the release of iron from transferrin and iron transport across the membrane by DMT1, a proton-coupled divalent metal transporter [13]. Iron-depleted transferrin (apotransferrin) is then recycled back to the cell surface where the restored physiologic pH 7.4 results in the dissociation of apotransferrin from TfR1. The entire cycle is completed within minutes and recurs 100–200 times in the duration of a single transferrin molecules life cycle in the body [14]. 

Iron in erythroid precursors is then transported to the mitochondria for heme synthesis. Heme itself functions as a transcriptional regulator. It can induce heme oxygenase 1, a molecule which reciprocally induces heme degradation [15]. Heme not participating in hemoglobin synthesis results in a downregulation of IRP2 which reduces TfR1 expression on the cell surface and thus the amount of iron entering cells [16]. Both of these functions prevent excess heme from accumulating in erythroid precursors. In order to prevent excess globin synthesis, heme deficiency represses globin synthesis by activating the heme regulated inhibitor which phosphorylates eIF2a and prevents the conversion of GTP from GDP, shutting down mRNA translation at the *β*-globin locus control region [17–19]. ALAS2, the first enzyme in the heme synthesis pathway, also has a 5′ untranslated region IRE, resulting in the reduction in heme synthesis in an iron deficient state. Heme itself can also repress ALAS synthesis in the liver [20]. Interestingly, during terminal differentiation of erythroid precursors, the regulation of ferritin and ALAS2 is disparate, with significant impairment of ferritin expression and undeterred ALAS2 translation [21]. The mechanism regulating this effect appears to preferentially channel iron for heme synthesis in erythroid precursors. These negative feedback mechanisms and reciprocal regulation of heme and iron transport during erythroid precursor regulation is essential to maintain all components in proper balance. 

Only a single molecule, FPN-1, has been identified to function as an iron exporter. FPN-1 is regulated by hepcidin. Additionally, FPN-1 mRNA has IREs on the 5′ untranslated region [22–24]. In iron deficiency, these two regulatory mechanisms predict opposite results; low hepcidin driving FPN-1 up but IRP binding to the IRE on the 5′ untranslated region would be expected to drive FPN-1 expression down. Hepcidin levels are low in iron deficiency while FPN-1 on the basolateral membranes of duodenal enterocytes is increased to absorb and transfer iron to circulating transferrin [25–27]. Because increased FPN-1 is observed in iron deficient mice, a non-IRE containing transcript of FPN-1 was hypothesized to enable iron absorption to proceed in the iron deficient state. Recently, an additional FPN-1 (FPN-1B) was discovered which lacks an IRE and is not subject to repression by IRP in iron deficiency [28]. FPN-1B is generated from an alternative upstream promoter and accounted for the increased expression of FPN-1 in the duodena of iron deficient mice. Furthermore, FPN-1B was also identified on erythroid precursors [28]. Why these cells need to export iron is not completely understood but it may enable erythroid precursors to sense systemic iron status and allow these precursors to respond to hepcidin levels. FPN-1B expression is diminished during the later stages of erythroid differentiation, that is once the erythroid precursor begins to produce hemoglobin, and possibly results in lower MCV and MCH during iron deficiency when hepcidin levels are low. 

Heme export has also recently been demonstrated in erythroid precursors. Based on data in cats, the function of feline leukemia virus, subgroup C receptor (FLVCR) was reported to be a heme exporter [29]. In infected cats, FLVC envelope protein induced blockade of FLVCR, leading to pure red cell aplasia due to a block of differentiation at the CFU-E or proerythroblast stage and resulting in reticulocytopenia and anemia [30, 31]. Studies show that all bone marrow cells are infected but the infection only results in an erythroid lineage phenotype, implying that FLVCR is uniquely important in erythroid development. FLVCR overexpression in mice results in a mild microcytic hypochromic anemia, suggesting that, since hypochromasia and microcytosis only result from heme or hemoglobin deficiency, FLVCR is needed to maintain heme and globin balance and avoid accumulation of free heme or excess globin in the cytoplasm [32]. The absence of FLVCR results in proerythroblast differentiation arrest and apoptosis, likely due to heme toxicity, and points to the significance of export of excess iron and iron-containing compounds. What happens to FLVCR during iron deficiency is yet to be determined but hepcidin expression in *F* *l* *v* *c* *r* ^−/−^ mice is increased with associated decrease in FPN-1 and a higher concentration of iron in the cell. 

Although hepcidin expression is expected to be low in iron deficiency, some clinical situations are more complex. Most recently, studies involving both human subjects (with iron refractory, iron deficiency anemia, or IRIDA) as well as mice (mask phenotype) present a situation in which high levels of hepcidin expression and iron deficiency coexist. These situations enabled scientists to uncover the role of membrane-bound serine protease type 6 (TMPRSS6) in the regulation of hepcidin during iron deficiency. TMPRSS6 is expressed in the liver. Mask mice have microcytic anemia due to iron deficiency caused by decreased iron absorption from high hepcidin levels. Positional cloning experiments uncovered the splicing error in *Tmprss6 *[33]. Similarly, patients with IRIDA have an autosomal recessive mutation in the gene encoding TMPRSS6 resulting in hypochromic microcytic anemia, low serum transferrin saturation, and inappropriately elevated hepcidin concentrations [34–37]. Recent studies suggest that TMPRSS6 normally acts to downregulate hepcidin expression by cleaving membrane-bound hemojuvelin (HJV) [37]. (See page 8 for a more extensive discussion of the regulation of hepcidin expression.)

## 3. Human Congenital Hypotransferrinemia

Transferrin is the main serum iron transporter in all vertebrates; it takes up iron from duodenal enterocytes where iron is absorbed and from macrophages when iron is recycled from senescent RBCs and delivers it to cells by binding TfR1. Congenital hypotransferrinemia is a rare hereditary disease characterized by a severe deficiency of serum transferrin. The defect results in iron deficient erythropoiesis and hypochromic anemia as well as iron overload in nonhematopoietic tissues (Figure 1). The excess iron is unavailable for erythropoiesis in this disease, suggesting that Tf-Fe(III) uptake by TfR1 is the only known means of iron delivery for erythropoiesis [38]. Fewer than a dozen cases have been described in the literature [39–44]. By analyzing case reports of this disease, we have learned that transferrin production occurs in excess of that required for normal erythropoiesis for which a minimum of only 10–20 mg/dL of transferrin is required [41], 20-fold less than levels typically found in circulation in normal individuals. The rarity of this disease in addition to the excess transferrin produced in normal individuals underscore the essential nature of this compound for normal physiologic function. 

Treatment for individuals diagnosed with congenital hypotransferrinemia involves the use of recurrent plasma infusions, plasma-derived human transferrin, or recombinant human transferrin. A case study reporting (1-2 g) transferrin infusions every 3-4 months resulted in patient improvement [39, 41]. This case demonstrates that effectiveness of a single dose of transferrin is between 4 and 9 months [41] despite a half life of only 7.6 days [14]. In another case, monthly plasma infusions provided sufficient transferrin to maintain a hemoglobin of 12 g/dL and enabled phlebotomy as simultaneous therapy for iron overload [42]. In this case, reticulocytosis was observed within 10–14 days of infusion followed by a rise in hemoglobin. Another case study reported the treatment of a boy with congenital hypotransferrinemia using monthly plasma infusion which resulted in a normal hemoglobin concentration [43, 44]. This last case provided amounts of plasma and patient hematocrit and enabled us to calculate some specific values.

 For example, if 300 mL of plasma was transfused, and plasma contains an average transferrin concentration of 200 mg/dL = 2 mg/mL, then 300 mL × 2 mg/mL = 600 mg transferrin per 300 mL plasma infusion. If total blood volume is approximately 5 L and hematocrit is roughly 30% (in an anemic patient), then plasma volume = 5000 mL × 0.7 = 3500 mL. 600 mg transferrin transfused into a volume of 3500 mL = 600 mg/35 dL = 17 mg/dL. With a *t*_1/2_ of 7.6 days, the end of 1 week results in <10 mg/dL of transferrin, the minimum concentration required to sustain erythropoiesis.

Because the circulating levels after one week are below minimum transferrin requirements for erythropoiesis, the efficacy of *monthly* plasma infusions implies that the amount of transferrin required for steady-state erythropoiesis is well below levels needed during stress erythropoiesis. In fact, the onset of a growth spurt at age 10 was associated with a drop in hemoglobin and an increase in reticulocytosis. The patient was treated with an increased rate of plasma infusions—weekly doses for two months—which lead to a rebound of hemoglobin and increased serum transferrin concentration approximately 3- to 5-fold from previously. Hepcidin excretion was negligible and increased over the course of two months of accelerated plasma infusions, suggesting that erythropoietic drive, as evidenced by worsening anemia and reticulocytosis, resulted in hepcidin suppression which was relieved by increasing iron supply to meet the demand of accelerated erythropoiesis [45]. 

A hypotransferrinemic (*hpx/hpx*) mouse model is described and characterized in the literature [46, 47] resulting from a splicing defect in the mouse transferrin gene on chromosome 9 [48]. Approximately 1% of normal transferrin protein is found in circulation in *hpx/hpx* mice [47]. These mice exhibit hypochromic microcytic anemia, low transferrin levels, severe growth retardation and a robust response to mouse plasma or purified transferrin injections, a pattern of characteristics reminiscent of human congenital hypotransferrinemia. Little iron is found in the bone marrow and spleen but massive iron overload develops in non-hematopoietic tissues such as the liver, heart, endocrine organs, and kidney [46, 47]. 

Although Tf-Fe(III) uptake occurs via binding to TfR1 in erythroid precursors (as well as other cell types), nontransferrin bound iron (NTBI), as in many diseases of iron overload, predominantly results in parenchymal iron deposition in non-hematopoietic cells. When transferrin levels are low, as in *hpx/hpx* mice, complete transferrin saturation occurs quickly. Once transferrin saturation approaches 100% and its iron binding capacity is exceeded, labile plasma iron (LPI) can be found in circulation [49]. LPI is a redox active form of NTBI which is taken up by cells in a dysregulated manner, can cause free radical damage resulting in the morbidity and mortality of iron overload diseases, and is unavailable for erythropoiesis. LPI is the presumed cause of iron overload in non-hematopoietic tissues in *hpx/hpx* mice.

Transferrin replacement in *hpx/hpx* mice relieves anemia, decreases parenchymal iron deposition in the liver, and reduces the high rate of iron absorption in the duodenum [50]. When transferrin-treated *hpx/hpx* mice are analyzed after transferrin concentration returns to undetectable levels, iron absorption increases despite persistent normal hemoglobin levels and decreased liver nonheme iron stores. This finding implies that transferrin concentration (or perhaps by extension transferrin saturation) itself has an effect on iron absorption, independent of the effect of hemoglobin concentration [50]. In fact, hepcidin expression, now known to control iron absorption, is under the regulation of Tf-Fe(III) [51] and hepcidin expression is low in *hpx/hpx* mice [52], resulting in increased iron absorption. In *hpx/hpx* mice, as in *β*-thalassemic mice, hepcidin levels do not reflect the degree of systemic iron overload, suggesting that a competing signal is counter-regulating hepcidin expression and provides further evidence for the existence of an “erythroid regulator” of hepcidin. 

The authors suggest that one week after transferrin injections, when transferrin levels have returned to baseline in *hpx/hpx* mice, “in spite of normalized hemoglobin levels…low transferrin levels lead to ineffective (iron-deficient) erythropoiesis.” [50] In fact, iron deficiency itself is associated with an increased rate of erythropoiesis and a preponderance of erythroid precursors that become quiescent without completing the maturation cycle [53]. In addition, *hpx/hpx* mice exhibit extramedulary erythropoiesis notably in the liver [47] and splenomegaly [46], findings reminiscent of *β*-thalassemia, a disease characterized by anemia, iron overload, and ineffective erythropoiesis. *Hpx/hpx* mice absorb somewhat more iron relative to *β*-thalassemic mice, which have a comparable anemia and reticulocytosis [54], suggesting that not the hemoglobin levels but the amount of transferrin itself, relative to the degree of erythropoietic demand, may influence hepcidin expression. In support of this, when *hpx/hpx* mice are transfused to suppress endogenous erythropoesis, the degree of iron absorption approached normal [55] possibly by reducing the pressure on Tf-Fe(III) delivery for erythropoiesis [50]. While further experimentation is necessary, these findings suggest that iron deficiency results in ineffective erythropoiesis as a consequence of relatively insufficient circulating transferrin to accommodate the degree of erythropoiesis [56].

## 4. *β*-thalassemia

*β*-thalassemias are caused by mutations in the *β*-globin gene resulting in reduced or absent *β*-chain synthesis. A relative excess of *α*-globin chain synthesis leads to increased erythroid precursor apoptosis, causing ineffective erythropoiesis which in turn results in extramedullary expansion and splenomegaly. Together with shortened RBC survival, these abnormalities result in anemia. The clinical phenotype is heterogeneous due to genotypically different mutations, combination inheritance with hemoglobinopathies, and additional modifying factors. Patients with *β*-thalassemia major, the most severe form of *β*-thalassemia, exhibit very limited synthesis of *β*-globin and require life-long RBC transfusions to ameliorate anemia and suppress extramedullary erythropoiesis. Without transfusions, expanded erythropoiesis results in hepatosplenomegaly and bone deformities due to expansion of the intraosseous marrow compartment. Patients with *β*-thalassemia intermedia show a milder clinical picture with more *β*-globin chains synthesized and require only intermittent transfusions. Although chronic transfusion therapy has been standard practice for the last half-century, advances in understanding the mechanisms of this disease have lead to some interesting findings and promise to continue evolving therapy in *β*-thalassemia.

Patients with *β*-thalassemia have increased intestinal iron absorption which, in addition to transfusion dependence, contributes to iron overload. If left untreated, iron overload results in progressive iron deposition, leading to multiple organ dysfunction and accounts for the majority of deaths in this disease. LPI has been proposed as the cause of this morbidity. As mentioned previously, because no physiologic means of iron excretion exist, increased iron absorption and RBC turn over from transfusion lead to saturation of plasma transferrin. When transferrin iron binding capacity is exceeded and NTBI is detectable in circulation, iron trafficking is dysregulated and results in the clinical manifestations of iron overload. Despite iron overload in patients with *β*-thalassemia, hepcidin levels are not increased. 

Insufficient hepcidin expression, relative to the degree of iron overload, is implicated as the cause of iron overload observed in *β*-thalassemia. We and others have demonstrated that hepcidin expression is relatively low for the degree of liver iron stores in untreated *β*-thalassemic mice relative to controls [57, 58]. Because hepcidin functions by binding FPN-1 and preventing iron release from duodenal enterocytes and macrophages, increased hepcidin levels are expected in diseases of iron overload to prevent continued iron absorption and release from stored recycled iron. In order to analyze the variability in hepcidin levels in *β*-thalassemic patients, serum samples were collected from patients and demonstrated that hepcidin levels increase in those with higher hemoglobin and decrease in those with high serum TfR1 levels, increased erythropoietin, and detectable NTBI [59]. These findings are consistent with other reports that correlate increased erythropoietic activity with hepcidin suppression as well as the response to increased hepcidin levels following transfusion. 

Several researchers have assessed the erythropoietic regulation of hepcidin from a clinical and basic science perspective. For example, transfusions in *β*-thalassemia major patients, used to suppress endogenous erythropoiesis, resulted in an increase in urinary hepcidin concentrations [60]. Furthermore, the exposure of HepG2 cells to sera from patients with *β*-thalassemia major after transfusion resulted in higher hepcidin levels relative to the cells exposed to sera of the same patients prior to the next transfusion [61, 62]. These findings suggest that hepcidin suppression in *β*-thalassemic patients results from the secretion of a soluble factor, the concentration of which is proportional to the degree of erythroid activity. This “erythroid regulator” of hepcidin has not yet been elucidated but is of great importance in diseases in which anemia and iron overload coexist. This signal is stronger than the regulation of hepcidin by iron and leads to the exacerbation of iron overload, the very complication associated with clinical deterioration and mortality in *β*-thalassemia.

Hepcidin suppression in diseases of iron overload with ineffective erythropoiesis exacerbates the degree of iron overload by increasing iron absorption. This excess iron deposits in the parenchyma of non-hematopoietic tissue. Because further iron absorption ultimately exceeds transferrin iron-carrying capacity, suppressed hepcidin results in the formation of NTBI which is not available for erythropoiesis. Prior experiments demonstrate that phlebotomy, erythropoietin administration, and hemolysis resulted in decreased hepcidin expression [26, 63, 64]. To examine the mechanism of hepcidin suppression in these conditions, separating the effect of erythropoiesis from that of anemia and iron stores is important. Ablation of erythropoiesis accomplished by chemotherapeutic agents, radiation, and erythropoietin-blocking antibodies prevents hepcidin suppression in response to hemolysis, bleeding, or epogen injection [64, 65]. Although ablation of erythropoiesis results in increased hepcidin expression, compensated hemolysis (without ablation of erythropoiesis) does not affect hepcidin expression despite an equivalent degree of anemia and hepatic iron deposition [64]. These studies further demonstrate that iron requirements for erythropoiesis influence hepcidin expression to a greater degree than anemia or non-hematopoietic iron stores in the body. 

New evidence regarding the influence of erythropoiesis on hepcidin levels involves growth differentiation factor 15 (GDF15) and twisted gastrulation 1 (TWSG1) in *β*-thalassemic patients and mice, respectively [66, 67]. These factors have recently been identified as possible soluble erythroid factors that regulate hepcidin [67]. GDF15 and TWSG1 levels are significantly elevated in *β*-thalassemic patients and mice, respectively. Sera from *β*-thalassemic patients with upregulated GDF15 suppress hepcidin mRNA expression in primary human hepatocytes, and depletion of GDF15 reverses hepcidin suppression. These findings suggest that increased serum GDF15 may be the factor through which ineffective erythropoiesis and/or increased erythroid precursor apoptosis influence hepcidin expression and iron homeostasis in *β*-thalassemic patients [66]. Alternatively, TWSG1, a protein synthesized at early stage of erythropoiesis, shows an indirect effect on hepcidin suppression [67]. It is possible that ineffective erythropoiesis in *β*-thalassemia modifies erythroid parameters such as GDF15 and TWSG1 which, together or through several different signaling pathways, induce inappropriate hepcidin inhibition and maldistribution of iron. 

Our laboratory hypothesized that this insufficient hepcidin secretion and maldistribution of iron in *β*-thalassemia may result from inadequate circulating transferrin to deliver iron for erythropoiesis. This hypothesis is informed by preliminary experiments in *H* *b* *b* ^th1/th1^ mice, a model of *β*-thalassemia intermedia, that demonstrates low non-heme iron in the bone marrow relative to control mice [57]. High-dose iron dextran in these mice results in a dose-dependent increase in extramedullary erythropoiesis. The lack of medullary erythroid response to iron suggests that increased transferrin concentration may be necessary to accommodate the degree of erythoid expansion observed in *β*-thalassemia. Chronic treatment with transferrin injections in *H* *b* *b* ^th1/th1^ mice results in increased hemoglobin production, decreased reticulocytosis and erythropoietin levels, reverses splenomegaly, and elevates hepcidin expression [56]. Transferrin injections also change the proportion of erythroid precursors to more mature relative to immature precursors, lower the rates of apoptosis in mature erythroid precursors, and reduce the amount of extramedullary erythropoiesis in the liver and spleen in *H* *b* *b* ^th1/th1^ mice. These findings imply that exogenous transferrin results in more iron delivery for erythropoiesis and restores more normal (less ineffective) erythropoiesis as a result. 

How additional transferrin is able to improve erythropoiesis is incompletely understood. One hypothesis may involve the observation that although the hemoglobin concentration and the number of RBCs increase after transferrin injections, RBCs are smaller and contain less hemoglobin. Similar phenotype of decreased MCV and MCH with normal hemoglobin values due to increased number of RBCs is observed in *T* *f* *R* ^+/−^ mice [68]. This lower MCV, both in our transferrin-treated *H* *b* *b* ^th1/th1^ mice and *T* *f* *R* ^+/−^ mice, is reminiscent of iron deficient erythropoiesis in normal subjects treated with recombinant erythropoietin [69]. This “functional iron deficiency,” a term used to describe states of iron restricted erythropoiesis induced by exogenous erythropoietin, applies also to states of ineffective erythropoiesis with elevated endogenous erythropoietin [70]. The underlying concept, that the rate of iron supply is insufficient to meet the demands of erythropoiesis, applies to other circumstances such as expanded erythropoiesis observed in *β*-thalassemia. The finding of protoporphyrin in RBCs of patients with *β*-thalassemia, in whom transferrin saturation is an ample 40%, provides evidence that this is the case [71]. In fact, old literature estimated that the daily iron requirement in *β*-thalassemia may be as high as 150 mg, values which could make iron demand by the expanded erythron greater than its available supply [72] and possibly trigger hepcidin suppression in order to stimulate an increase in iron absorption.

## 5. Hereditary Hemochromatosis

Hereditary hemochromatosis (HH) is a genetically inherited disorder of iron metabolism. Although gene frequency is as high as 5–7%, low penetrance results in only 1:300 to 1:400 affected individuals. Four types of disorders exist, all of which result in increased intestinal iron absorption as a consequence of inadequate hepcidin, or hepcidin insensitivity, relative to the degree of systemic iron (Table 1). 

**Figure 2:**
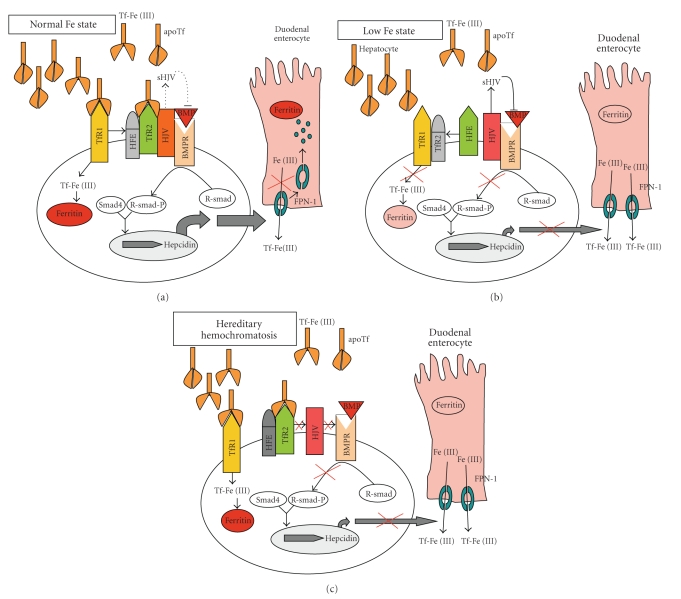
Hepcidin regulation through the BMP pathway, demonstrating effects of a functional response to systemic iron status and the dysregulation of proteins resulting in iron overload.

The most common type of HH, Type I HH, is characterized by a mutation in the HFE gene. Homozygous C282Y mutation, which accounts for more than 80% HH patients [73], disturbs formation of a disulfide bond in the *α*3 domain of HFE, prevents its binging to *β*2-microglobulin—a protein involved in the regulation of iron absorption, and markedly reduces the appearance of HFE on the cell surface. Disease is occasionally present as a compound heterozygote mutation with a second synergistic H63D mutation which also leads to the reduction of cell surface HFE [74]. Although the molecular function of HFE remains uncertain, recent evidence points to its role in hepcidin regulation [75]. Because hepcidin is believed to be the central regulator of iron flows in the body, the involvement of HFE in hepcidin regulation is consistent with its presumed function in iron absorption. In the recent past, major advances have been made in understanding the molecular mechanism of hepcidin regulation. The bone morphogenic protein (BMP) pathway—involved in cell proliferation, differentiation, and apoptosis—has been identified as a critical regulator of hepcidin expression [76, 77]. BMP receptor activation results in Smad phosphorylation which translocates to the nucleus and activates transcription of target genes [78] (Figure 2). Hemojuvelin (HJV) was identified as an iron-specific BMP coreceptor and stimulant of the BMP pathway in iron overload states [79]. Another form of HJV—soluble HJV (sHJV)—acts as a BMP antagonist [80] and leads to reduced hepcidin expression [77] as a means of negative feedback (Figure 2).

Expression of hepcidin is inappropriately low in patients with Type I HH and *H* *f* *e* ^−/−^ mice [81–83]. HFE is one of several membrane proteins involved in communicating systemic iron status to the hepatocyte and affects HJV/BMP signaling pathway to positively influence hepcidin production (Figure 2). Suppressed HFE levels in HH prevent appropriate sensing and result in a dampened hepcidin response to iron load, consequent increased iron absorption and increased transferrin saturation. *H* *f* *e* ^(−/−)^ mice confirm the association of HFE gene mutation, iron overload, and increased erythropoiesis. Compared to wild type, *H* *f* *e* ^−/−^ mice exhibit massive iron deposition and elevated transferrin saturation [84]. The role of HFE in influencing iron absorption is not completely understood. HFE associates with TfR1 under low iron conditions [85] and is displaced when TfR1 binds Tf-Fe(III) [86–88] (Figure 2). Unlike TfR1, TfR2 lacks an IRE and is thus not regulated by levels of plasma iron. As serum iron concentration increases, TfR2 expression exceeds that of TfR1 and Tf-Fe(III) binds both TfR1 and TfR2, increasing TfR2 stability [89, 90] on the membrane and induces HFE binding to TfR2. This HFE/TfR2 complex interacts with HJV, the iron-specific BMP co-receptor, and potentiates the BMP signaling pathway and hepcidin transcription [91] (Figure 2).

Thus, both TfR2 and HFE/TfR1 complex function as the main Tf-Fe(III) sensors [90, 92] and communicate systemic iron status to the hepatocyte resulting in altered hepcidin secretion. As expected, iron overload diseases are also associated with mutation in genes coding these intermediary proteins. Type II HH results from HJV or hepcidin mutations. As those with HFE mutations, patients with mutated HJV/HAMP also exhibit enhanced iron absorption and rapid iron accumulation at a young age [93]. *HJV* mutations, like mutations in *hepcidin* itself, result in nearly absent hepcidin expression and clinically result in the most severe form of HH termed Juvenile Hemochromatosis. *H* *j* *v* ^−/−^ mice develop a substantial decrease in hepcidin expression with concurrently increased FPN-1 expression and early onset iron deposition [94]. Type III HH results from a mutation in the gene encoding TfR2. TfR2 depletion leads to hepatic iron deposition and decreased hepcidin expression in zebrafish embryos [95]; mice models exhibit elevated ferritin and transferrin saturation in addition to these features [96]. 

Lastly, type IV or FPN-1 mutation is an autosomal dominant HH. Because mutations in the gene encoding FPN-1 may affect its presentation on the cell membrane as well as its ability to bind hepcidin, there are two clinical features of this genetic disorder [97]. When FPN-1 mutation leads to reduced FPN-1 surface expression (*loss-of-function* mutation), patients develop low transferrin saturation and Kupffer cell iron loading due to limited iron export function and become anemic when treated with phlebotomy. These patients are unable to mobilize their iron stores due to mutant FPN-1. The *flatiron* mouse, with a heterozygous missense mutation in FPN-1, demonstrates iron loading of Kupffer cells, high serum ferritin, and low transferrin saturation, similar to patients with classic “ferroportin disease” described above [98]. Although FPN-1 on duodenal enterocytes is likely also affected, the export of 2–4 mg of iron during iron absorption may be easier to accomplish despite the mutation than the 20 mg of iron recycled daily by macrophages [99]. 

The other type of FPN-1 mutation results in normal FPN-1 cell surface expression with functional iron export and is characterized by insensitivity to hepcidin (*gain-of-function* mutation). This mutation is associated with high transferrin saturation and hepatocyte iron loading [100]. As proof of principle, De Domenico developed an FPN-1 mutated cell line that shows a normal iron efflux activity but does not respond to increased hepcidin [99]. This lack of response of mutated FPN-1 to hepcidin is associated with increased duodenal iron absorption, increased transferrin saturation, and increased iron deposition in patient hepatocytes. The phenotype of “hepcidin-resistant” HH is similar to the hepcidin deficient phenotype in other types of HH. 

Overall, HH mutations are associated with hepcidin suppression or hepcidin insensitivity. Hepcidin injections inhibit the increased iron absorption in the duodena of *H* *f* *e* ^−/−^ mice [101] and forced expression of hepcidin corrects the hemochromatosis phenotype [83]. Unlike in *β*-thalassemia, sera from patients with HH do not result in hepcidin suppression in HepG2 cells [102] and induce an increased hepcidin gene expression in normal hepatocytes [61]. These findings demonstrate mutant HFE on hepatocytes results in insufficient hepcidin stimulation in HH patients leading to increased iron absorption and could be the major reason for iron overload in this disease. 

Increased hemoglobin, MCV, and MCH have been demonstrated in patients with HH [103] and *H* *f* *e* ^−/−^ mice [84], suggesting that increased iron absorption influences erythropoiesis. A systematic analysis of erythroid parameters in HH has not been reported. In one study, 94 patients homozygous for C282Y were analyzed and found to have a 7–9% increase in hemoglobin, MCV, and MCH compared to normal controls; no increase in RBC count was observed [103]. This represents approximately 5 g of additional hemoglobin and enabled these individuals to sequester 170 mg of iron. This net increase in the heme and hemoglobin synthesis is not completely understood since normal erythroid precursors control heme synthesis by exerting a negative feedback mechanism on ALAS by heme itself [20]. However, ALAS2, with a 5′ untranslated region IRE, may result in continued heme synthesis during iron overload. This compensation to safely sequester iron by increasing hemoglobin, MCV, and MCH above normal levels requires additional experimentation to more completely understand the mechanisms of physiologic set points in iron utilization for hemoglobin synthesis.

## 6. Summary

Erythropoiesis and iron metabolism must be closely coordinated to ensure adequate supply of iron for erythropoiesis. Conversely, the inability to prevent excess production of all hemoglobin intermediates including iron, heme, and globin is hazardous and results in its own pathologic consequences. Hepcidin is a key regulator of iron absorption and recycling and is itself under the regulation by erythropoiesis as evidenced by its suppression in diseases of ineffective erythropoiesis despite systemic iron overload. Transferrin and TfR1/2 are intimately involved in the regulation of hepcidin; the dysregulation of these components results in the inappropriate trafficking of iron in the organism. How sensing of the body's iron status occurs is still incompletely understood although it is becoming clear that the total systemic iron is not as important as iron availability for erythropoiesis. A tremendous degree of reserve is built into the system to accommodate for minor excesses and deficiencies and the physiology of the “normal state” is uncovered only when the pathology exceeds the body's ability to compensate by using these reserves. The distribution of iron between cells is dependent on the amount of total available iron but also relies on the ability of the body to deliver that iron for erythropoiesis. Greater understanding of the interplay between the key factors involved in the regulation of iron metabolism and erythropoiesis will help develop more effective therapies for disorders of iron overload, iron deficiency, and hemoglobin synthesis.

## Figures and Tables

**Table 1:** Characteristics of Hereditary Hemochromatosis.

	Gene	Mutation	Inheritance	Hepcidin levels	Pathophysiology
Type I	HFE	C282Y (6p21), H63D	AR	Low	Increased iron absorption
Type II –Juvenile hemochromatosis	HJV; hepcidin	1q21; 19q13	AR	Low	Increased iron absorption
Type III	TFR2	7q22	AR	Low	Increased iron absorption
Type IV*	FPN1	2q32	AD	High	Increased iron absorption

*Mutation results in 2 similar forms of disease: either a hemochromatosis-like illness with increased iron in hepatocytes due to hepcidin resistance (*gain-of-function* mutation) or reduced macrophage iron export with normal transferrin saturation called “classic ferroportin disease” (*loss-of-function* mutation).
